# Dual mechanism of anti-seizure medications in controlling seizure activity

**DOI:** 10.1093/braincomms/fcag088

**Published:** 2026-03-16

**Authors:** Guillermo M Besné, Emmanuel Molefi, Billy Smith, Nathan Evans, Sarah J Gascoigne, Chris Thornton, Fahmida A Chowdhury, Beate Diehl, John S Duncan, Andrew W McEvoy, Anna Miserocchi, Jane de Tisi, Matthew C Walker, Peter N Taylor, Yujiang Wang

**Affiliations:** CNNP Lab (www.cnnp-lab.com), School of Computing, Newcastle University, Newcastle upon Tyne NE4 5TG, United Kingdom; CNNP Lab (www.cnnp-lab.com), School of Computing, Newcastle University, Newcastle upon Tyne NE4 5TG, United Kingdom; CNNP Lab (www.cnnp-lab.com), School of Computing, Newcastle University, Newcastle upon Tyne NE4 5TG, United Kingdom; CNNP Lab (www.cnnp-lab.com), School of Computing, Newcastle University, Newcastle upon Tyne NE4 5TG, United Kingdom; CNNP Lab (www.cnnp-lab.com), School of Computing, Newcastle University, Newcastle upon Tyne NE4 5TG, United Kingdom; CNNP Lab (www.cnnp-lab.com), School of Computing, Newcastle University, Newcastle upon Tyne NE4 5TG, United Kingdom; Department of Clinical and Experimental Epilepsy, UCL Queen Square Institute of Neurology, University College London, London WC1N 3BG, United Kingdom; Department of Clinical and Experimental Epilepsy, UCL Queen Square Institute of Neurology, University College London, London WC1N 3BG, United Kingdom; Department of Clinical and Experimental Epilepsy, UCL Queen Square Institute of Neurology, University College London, London WC1N 3BG, United Kingdom; Department of Clinical and Experimental Epilepsy, UCL Queen Square Institute of Neurology, University College London, London WC1N 3BG, United Kingdom; Department of Clinical and Experimental Epilepsy, UCL Queen Square Institute of Neurology, University College London, London WC1N 3BG, United Kingdom; Department of Clinical and Experimental Epilepsy, UCL Queen Square Institute of Neurology, University College London, London WC1N 3BG, United Kingdom; Department of Clinical and Experimental Epilepsy, UCL Queen Square Institute of Neurology, University College London, London WC1N 3BG, United Kingdom; CNNP Lab (www.cnnp-lab.com), School of Computing, Newcastle University, Newcastle upon Tyne NE4 5TG, United Kingdom; Department of Clinical and Experimental Epilepsy, UCL Queen Square Institute of Neurology, University College London, London WC1N 3BG, United Kingdom; Faculty of Medical Sciences, Newcastle University, Newcastle upon Tyne NE1 7RU, United Kingdom; CNNP Lab (www.cnnp-lab.com), School of Computing, Newcastle University, Newcastle upon Tyne NE4 5TG, United Kingdom; Department of Clinical and Experimental Epilepsy, UCL Queen Square Institute of Neurology, University College London, London WC1N 3BG, United Kingdom; Faculty of Medical Sciences, Newcastle University, Newcastle upon Tyne NE1 7RU, United Kingdom

**Keywords:** neurophysiology, dynamic states, seizure states, seizure propagation, bifurcation

## Abstract

Anti-seizure medications (ASMs) can reduce seizure duration, but their precise modes of action are unclear. Specifically, it is unknown whether ASMs shorten seizures by curtailing existing seizure activity early or by selectively suppressing certain seizure activity patterns from emerging. We retrospectively analysed intracranial EEG (iEEG) recordings of 457 seizures from 28 people with epilepsy undergoing ASM tapering. Beyond measuring seizure occurrence and duration, we categorized distinct seizure propagation activity patterns (states) based on spatial and frequency power characteristics and related these to different ASM levels. We found that reducing ASM levels led to increased seizure frequency (*r* = 0.87, *P* < 0.001) and longer seizure duration (*β* = −0.033, *P* < 0.001), consistent with prior research. Further analysis revealed two distinct mechanisms in which seizures became prolonged: Emergence of new seizure propagation patterns—In ∼40% of patients, ASM tapering unmasked additional seizure activity states, and seizures containing these ‘taper-emergent states’ were substantially longer (*r* = 0.49, *P* < 0.001). Prolongation of existing seizure patterns—Even in seizures without taper-emergent states, lower ASM levels still resulted in ∼12–224% longer durations depending on the ASM dosage and tapering (*β* = −0.049, *P* < 0.001). ASMs influence seizures through two mechanisms: they (i) suppress specific seizure propagation patterns (states) in an all-or-nothing fashion and (ii) curtail the duration of other seizure patterns. These findings highlight the complex role of ASMs in seizure modulation and could inform personalized dosing strategies for epilepsy management. These findings may also have implications in understanding the effects of ASMs on cognition and mood.

## Teaser

Anti-seizure medications (ASMs) are known to reduce seizure duration, but there appears to be a dual mechanism through which this is achieved. First, ASMs suppress specific seizure activity and propagation patterns in an all-or-nothing fashion depending on dosage and (ii) curtail the duration of other seizure patterns.

## Graphical Abstract

**Graphical Abstract:**
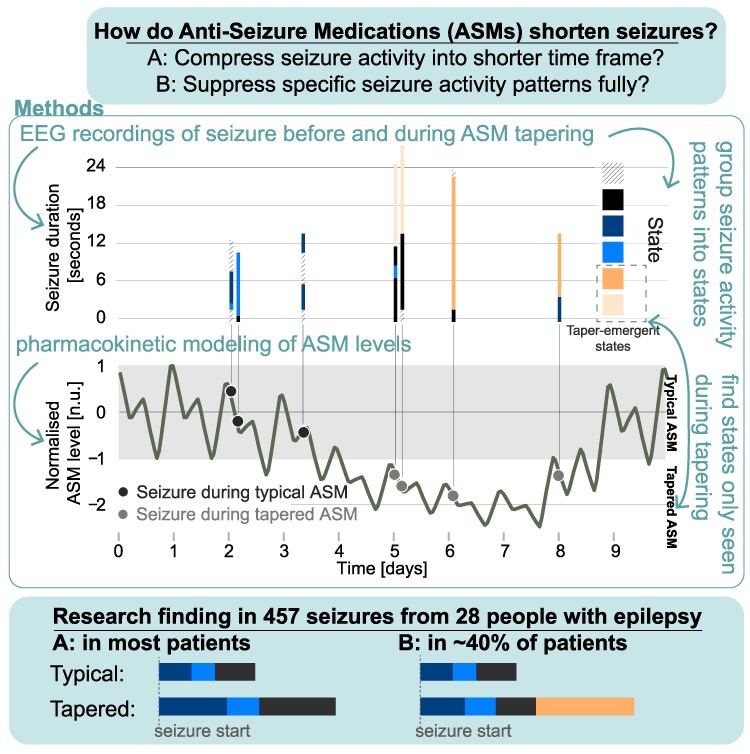

## Introduction

Anti-seizure medications (ASMs) are a cornerstone of epilepsy treatment, helping many patients achieve seizure control.^1^ However, determining the right dosage remains a challenge, as treatment must balance seizure suppression with potential side effects. Currently, ASM dosing follows a trial-and-error approach, tailored to each patient’s unique response. While seizure suppression is generally dose-dependent, ASMs can reach a ceiling effect, beyond which increasing the dose provides no additional benefit.^2^ In fact, ∼30% of epilepsy patients continue to experience seizures despite trying multiple ASMs at increasing doses.^3^ Although the molecular and cellular mechanisms of ASMs have been well studied,^1,4-7^ their dose-dependent effects on human brain activity and seizure dynamics *in vivo* remain poorly understood.

Previous studies suggest that higher ASM doses are associated with shorter seizures.^7-9^ However, the mechanisms underlying this effect are unclear—ASMs may simply curtail seizure activity early, or actively suppress the involvement of specific brain regions and activity patterns in an all-or-nothing fashion. In this study, we tested if either of these two mechanisms dominate, or if a mixture of both mechanisms exist.

To this end, we retrospectively analysed intracranial EEG (iEEG) data from patients undergoing pre-surgical epilepsy monitoring, where ASMs are often gradually withdrawn to trigger more severe seizures. This controlled setting provides a unique opportunity to investigate how different ASM doses and combinations influence brain activity in humans *in vivo*. Building on previous work,^10,11^ we dissect seizure activity into activity patterns and therefore describe each seizure as a sequence of these patterns or states. By examining seizure states across varying ASM levels, we aim to uncover fundamental principles of drug action that may be relevant across neurological disorders, and may contribute to precision medicine approaches.

## Materials and methods

### Subject and study information

In this retrospective study, we included 28 subjects with medically refractory epilepsy who underwent intracranial EEG (iEEG) monitoring and medication tapering. Anonymized ictal recordings were obtained from the National Epilepsy & Neurology Database and analysed with the approval of Newcastle University Ethics Committee (42569/2023). All available subjects from the National Epilepsy & Neurology Database for this study were contributed by the National Hospital for Neurology and Neurosurgery (NHNN).

For this study, we only included subjects for whom we had at least three usable seizure EEG and imaging data to allow for localizing iEEG contacts to brain regions. Electrographic seizure onset and offset times were marked by expert epileptologists at the NHNN, algorithmically verified, and visually confirmed by SJG, NE, and YW. If seizures occurred in quick succession (i.e. <120 s between offset and subsequent onset), only the lead seizure was included. We further excluded any seizures following sleep deprivation or cortical stimulation procedures, or occurring during periods where ASM levels exceeded the typical range (e.g. following administration of rescue medication).

### iEEG pre-processing

All seizure EEG recordings were extracted with an accompanying 120 s pre-ictal period. Any EEG sampled at higher than 512 Hz was resampled with anti-aliasing to 512 Hz. All iEEG signals were then band-pass filtered between 0.5 and 200 Hz, and notch filtered at 50 Hz (and harmonics) using a 2 Hz window to exclude line noise. All filtering was performed using a zero-phase response 4-th order Butterworth filter. To identify pre-ictal noise, an iterative detection algorithm was used, followed by visual validation. For each subject, noisy iEEG channels were removed from all seizures. If a single seizure had many noisy channels, we opted to exclude that seizure to maintain the maximum number of channels for that subject. Finally, recordings were re-referenced to a common average.

### Localization of electrodes to Regions of Interest

iEEG electrode contacts were localized to brain regions of interest (ROIs) to provide anatomical context. Following our previously described pipeline,^12,13^ electrodes were mapped to 120 ROIs from the Lausanne ‘scale60’ atlas^14^ using pre-operative MRI parcellations generated via FreeSurfer.^14,15^ Electrode contacts were assigned to the nearest grey matter region within 5 mm, with those beyond this distance excluded from analysis. For further detail on the pipeline used in this study, see Woodhouse *et al*.^16^

### Identification of seizure states

**Figure 1:**
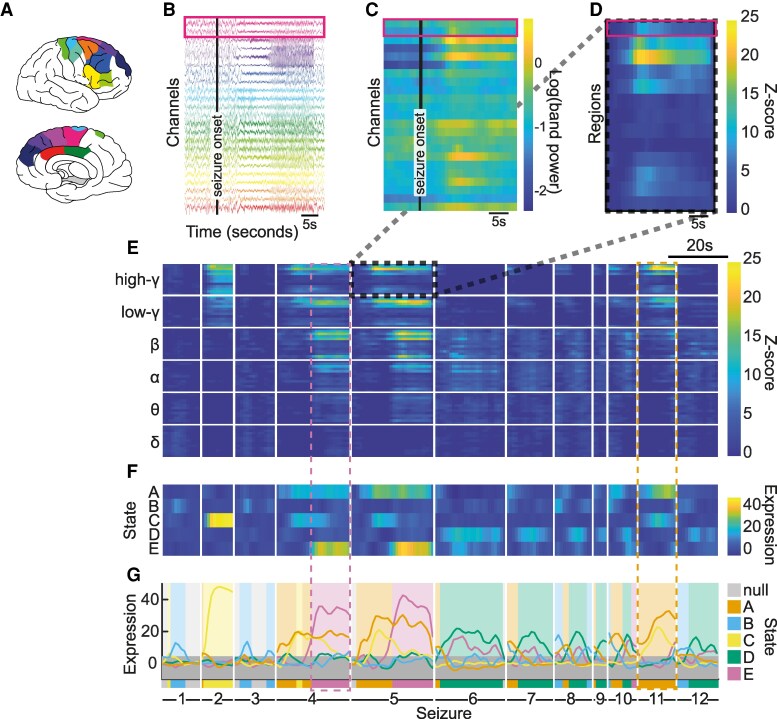
**Seizure state identification from EEG for one example subject.** (**A**) and (**B**) Implanted brain regions and associated EEG channels. Brain schematic are produced with the Simple-Brain-Plot toolbox (https://github.com/dutchconnectomelab/Simple-Brain-Plot). (**C**) Log power in high *γ* band in overtime windows of 5 s with 4 s overlap in all EEG channels. (**D**) Z-scored log power of ictal versus pre-ictal period in high *γ* band averaged within each brain region. (**E**) Data matrix *X* consisting of z-score matrices stacked across frequency bands and concatenated across seizures. (**F**) Expression matrix *H* following NMF of *X*. (**G**) State identification using dominant component in *H* at each time window. Note that panel (**E**) is a time-frequency representation of each seizure, and we have highlighted with two dashed boxes the time-frequency representations for two states A and E.

To characterize subject-specific seizure activity patterns, we grouped time periods within all seizures of an individual into ‘seizure states’.^10,11^ Fig. 1 demonstrates the workflow from iEEG (panel A and B) to state allocation (panel G). Supplementary Section 2 provides additional algorithmic details.

For each seizure in every subject, we computed band power using a 5 s sliding window with 4 s overlap in six distinct frequency bands (*δ*: 1–4 Hz, *θ*: 4–8 Hz, *α*: 8–13 Hz, *β*: 13–30 Hz, low-*γ*: 30–60 Hz, and high-*γ*: 60–100 Hz). Figure 1A and B show an example seizure for an example subject, with implanted brain regions colour-coded. Band power data were log-transformed (Fig. 1C shows the high *γ* band), and then averaged across recording channels in each brain region. We *z*-scored band power values from seizure time windows to the pre-ictal period (Fig. 1D). Thus, for each seizure, and each frequency band, we obtained a matrix with dimensions $n_{\mathrm{regions}}\times n_{T}$, where $n_{\mathrm{regions}}$ and $n_{T}$ denote the number of recorded regions, and of time windows in a given seizure, respectively.

For each subject, we then stacked and concatenated all such matrices to create data matrix *X* (Fig. 1E) with dimensions $(n_{\mathrm{regions}}\times n_{\mathrm{bands}})\times n_{T_{\mathrm{all}}}$, where $n_{\mathrm{bands}}=6$ is the number of frequency bands, and $n_{T_{\mathrm{all}}}$ the number of time-windows across all seizures in a given subject.

To obtain seizure states, we used non-negative matrix factorization (NMF) to decompose *X* into two non-negative matrices (X≈W×H), such that the information in *X* is summarized by a few components. Here, how strongly each component is expressed at any given time window is captured in *H*, which has dimensions $n_{c}\times n_{T_{all}}$ with $n_{c}$ being the number of components. Figure 1F shows *H* with $n_{c}=5$ in our example subject. *W* has dimensions $(n_{\mathrm{regions}}\times n_{\mathrm{bands}})\times n_{c}$ and captures the patterns of seizure activity in terms of frequency band and brain regions. We can interpret these as spatio-frequency patterns of seizure activity. Together, *W* tells us what patterns of seizure activity are present, and *H* tells us how strongly these are expressed at any given time window.

We used *H* to assign each time window to a component (denoted herein as ‘state’) by selecting the most strongly expressed component. This simplistic approach effectively turns the soft-clustering provided by NMF into a hard clustering, and we added some thoughts for future work in our Discussion on this choice. In some time windows, none of the components are particularly strongly expressed, indicating that there is generally a low level of seizure activity. We therefore thresholded the expression level (Fig. 1G, grey area) and assigned time windows without strong expression to a ‘null’ state. This step means that we are primarily capturing seizure propagation patterns, and ignoring subtle seizure onset patterns (see Discussion). In Fig. 1G, we show the final identified states in all seizures in our example subject.

As a technical note, these seizure states are best understood as ‘propagation’ patterns. Seizure onset is unlikely to feature as its own state due to the limited temporal and spatial activation.

### ASM tapering and plasma concentration estimation

The modeling of ASM plasma concentration described in our previous study^17^ is replicated here with some modifications. Each subject’s ASM dosage schedule was obtained from clinical records then converted into a continuous estimation of plasma concentration using known pharmaco-kinetics.^8,18-20^ It is important to note that the precise timing of each intake is not available, and provided is only the schedule of intake. To retain the natural daily fluctuations of the medication and reduce the effects of this lack of accuracy, we applied a 6 h moving average with a 5 h overlap.

**Figure 2:**
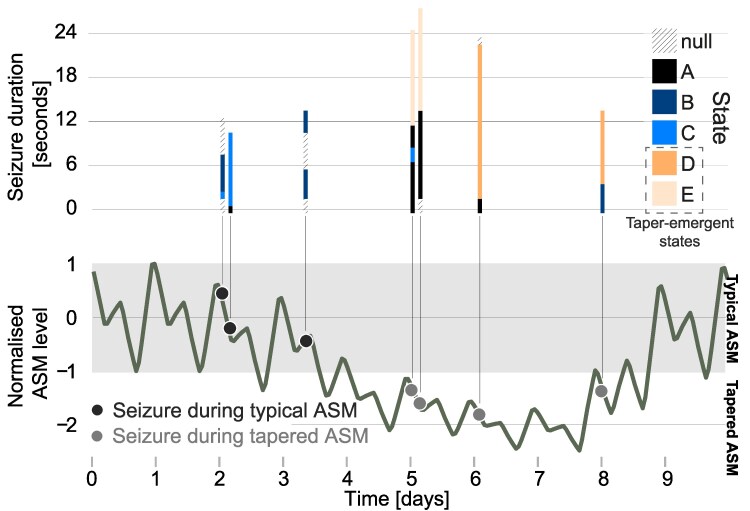
**ASM level and seizure states for an example subject.** Top: Seizure states for each seizure are plotted as a colour-coded bar in time. Y-axis indicates duration of each seizure. Bottom: Normalized ASM levels over recording time with the ‘typical’ ASM condition (between ± 1) highlighted in grey. Seizures are marked as dots and aligned with top panel. Seizure states D and E are ‘taper-emergent states’ as they only appear in seizures that occurred during the tapered ASM condition. Each data point represents a seizure.

The variability in pharmacokinetics and dosage, results in a different range of blood plasma concentration (BPC) across subjects and ASMs. However, the range of fluctuations before tapering should have similar therapeutic effect across subjects.^8,17,21^ To allow for comparison, we normalized plasma concentration values into a single ‘ASM level’ that would fluctuate between ± 1 during non-tapered (typical ASM) periods for each subject. Initially, each ASM plasma concentration was normalized individually using


(1) $$\mathrm{BP}C_{\mathrm{norm}}=\frac{\mathrm{BPC}-\mathrm{mean}(\mathrm{BP}C_{\mathrm{steady}})}{\mathrm{range}(\mathrm{BP}C_{\mathrm{typical}})/2},\;$$

where $\mathrm{BP}C_{\mathrm{typical}}$ is BPC extracted from the non-tapered typical medication regime of the subjects at the start of the recording. A combined plasma concentration was then calculated as a mean of all medications a subject was taking and re-normalized using the same equation, i.e. Eq. 1. Figure 2 (bottom panel) presents the resultant normalized ASM level estimation in an example subject. This normalized ASM level will be used throughout the remaining paper.

We then algorithmically defined ‘typical’ and ‘tapered’ ASM periods in each subject according to thresholds: Typical periods represent the expected ASM levels according to the subject’s treatment regimen prior to tapering. Periods with ASM levels in the range of ± 1 were thus labelled as ‘typical’. We labelled periods with ASM levels below −1 as ‘tapered’. As previously mentioned, periods where ASM levels exceeded a value of 1 (rescue medication) were excluded. Figure 2 (bottom panel) provides a visual summary of this definition in an example subject.

Finally, within each subject, we identified seizure states that only occurred in seizures during the tapered condition (if any existed), and term them ‘taper-emergent states’. These represent seizure activity patterns in certain frequency bands and brain regions that are only seen in seizures during the tapered condition. Figure 2 (top panel) shows this in our example subject. Note that in contrast to these ‘taper-emergent states’, we refer to all other states in a given subject as ‘dose-independent states’ given these can occur independent of ASM condition.

### Statistical analyses

For our analysis, we performed a log10 transformation of seizure duration, determined from clinically labelled seizure onset and offset times. This choice was guided by previously described methods^8,11^ due to the distribution of seizure duration generally being heavy-tailed. The log transformation effectively means that we are comparing relative seizure duration. For example, if seizure log duration decreased by −0.2 between two conditions, this can be translated as a 63% ($10^{-0.2}=0.63$) reduction to duration. We report these percentage reductions throughout the Results.

Using the whole dataset, we compared seizure frequency and duration across different ASM levels. Seizure frequencies during typical and tapered periods were compared using a paired-sample Wilcoxon signed-rank test. To compare seizure categories (that is, seizures without taper-emergent states from both the tapered and typical conditions, and seizures with taper-emergent states only observed in the tapered condition) in seizure duration data, we used the Wilcoxon rank-sum test. We report the *r*-value and associated *P*-value.

We then related the continuous measures of ASM level and duration of seizures using a linear mixed-effects model (LMM) with the following formula:


(2) $$\begin{array}{rl} \log\text{-}\mathrm{duration}\;∼\;\beta_{0\;} & +\beta_{1}\mathrm{ASM}\text{-}\mathrm{level}+\beta_{2}\sin\;(24\mathrm{hT})\\ & +\;\beta_{3}\cos\;(24\mathrm{hT})+\beta_{4}\sin\;(12\mathrm{hT})\\ & +\;\beta_{5}\cos\;(12\mathrm{hT})\\ & \begin{array}{c} +\;(1|\;\mathrm{subject}) \end{array} \end{array}$$

where subject is included as a random intercept to control for systematic differences in seizure duration between subjects. Because seizure durations have been reported to have circadian and ultradian rhythms,^22,23^ we have included circular variables (sine and cosine of 12hT and 24hT) to ensure that the effects modelled are not simply capturing 12 or 24 h fluctuations in seizure durations. We report the slope coefficient for ASM-level ($\beta_{1}$) and the associated *p*-value as a measure of the relationship between ASM-level and seizure duration, after factoring out subject-specific and chronobiological effects.

To test for group-level effect of, e.g. tapered versus typical ASM conditions on seizure duration, we compared LMM-corrected durations of typical and tapered seizures using Wilcoxon rank-sum tests. The LMM-corrected durations are simply the residuals of Eq. 2 with the ASM-level effect added back. These corrected durations represent the duration of seizures after removing subject-specific offsets and chronobiological effects.

Throughout this work, we provide *P*-values as a reference. However, we do not use these *P*-values for down-stream analyses, and avoid the interpretation of statistical significance.

## Results

### Subject and data characteristics

In this retrospective study, we assessed 457 seizures in 28 adult subjects who underwent iEEG monitoring and ASM tapering. Our cohort contained a mixture of temporal lobe and extra temporal epilepsies. Full metadata, ASM intake and tapering data, as well as demographics are provided in Supplementary Section 1.

### ASM tapering triggers more frequent and longer seizures

**Figure 3:**
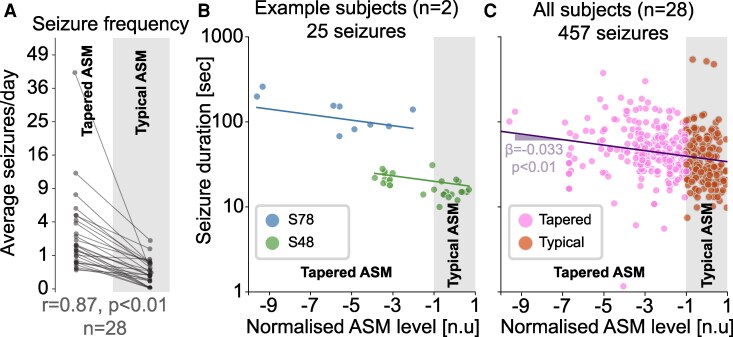
**Seizure frequency and duration change with ASM tapering.** (**A**) Seizure frequency measured as number of seizures per day averaged across the entire condition (‘Typical ASM’ or ‘Tapered ASM’) is shown for all subjects (n=28). Each data point represents the average seizure frequency per day for one subject calculated in the two ASM conditions. Wilcoxon’s signed-rank test shows a substantial increase (r=0.87, p<0.001) in seizure frequency in the tapered condition. (**B**) In two example subjects, we plotted seizure duration against normalized ASM levels in all their seizures. Lines of best fit are plotted for each subject for reference. Each data point represents a seizure. (**C**) For all subjects (n=28) and their 457 seizures, we plotted seizure duration after removing systematic differences between subjects (using a random offset LMM) against normalized ASM levels. Seizures occurring in the typical and tapered conditions are shown in orange and pink, respectively. Best fit of fixed effects from a LMM is shown as a solid line, with a slope β=−0.033, p<0.001. Each data point represents a seizure.

First, to confirm findings from previous literature and validate our data, we investigated whether ASM tapering was associated with a change in seizure frequency, and duration. We compared seizure frequency, measured as seizures per day, between typical (normal ASM dosage) and tapered conditions (Fig. 3A). Seizure frequency was substantially higher in the tapered condition than in the typical condition (Wilcoxon signed-rank test r=0.87, P<0.001).

Finally, we asked whether seizure log-duration was correlated with ASM withdrawal. Figure 3B displays the log-duration against normalized ASM plasma concentrations for two example subjects. It is clear from Fig. 3B that different subjects have systematically different seizure durations (as reported by Ghosn *et al.*^8^), which requires a subject-level normalization to enable group-level comparisons. To this end, we used a LMM with subject-level random intercepts. This allowed us to put all subjects on the same scale in terms of duration as shown in Fig. 3C. Our analysis using a LMM showed a strong link between ASM withdrawal and longer seizure durations (β=−0.033, P<0.001). The value β=−0.033 represents the change in log10 (seizure duration) for each unit change in normalized ASM levels. This translates to an ∼8% increase in seizure duration for every unit reduction in normalized ASM levels. Under typical conditions (with a stable ASM dosage), ASM levels naturally fluctuate between +1 and −1 units. This means that within this normal range, seizure duration may vary by ∼16%. However, during ASM tapering, each unit decrease in ASM levels is expected to further increase seizure duration by about 8%. Most subjects in our study experienced a 2- to 10-fold reduction in normalized ASM levels. Thus, we estimate that seizure duration could additionally increase by 16% to 80% in the tapered condition. Full results of this model are presented in Supplementary Section 3. We also report data summaries by ASM type in Supplementary Section 1, but avoided a formal analysis due to complex effects of poly-therapy and selective ASM tapering. We did not observe clear evidence of a selective effect by any individual ASM type.

Having confirmed that in our dataset, seizure duration is indeed increased with ASM tapering, we then sought to investigate how exactly the seizure EEG patterns change with ASM tapering.

### ASM tapering reveals new seizure propagation patterns

To understand the exact EEG patterns, we divided all the seizure EEG patterns for a given subject into a distinct number of seizure states (see Materials and Methods). This usually decomposed each seizure into a sequence of a few states (Fig. 1), which represent their spatio-temporal propagation patterns on EEG.

We first explored if some states (i.e. seizure EEG patterns) only appear in the tapered condition within each subject. To achieve this, we selected subjects who had at least three seizures overall, with at least one in each of the tapered and typical ASM conditions, resulting in 304 seizures across 17 subjects. This selection step ensured that, per subject, we saw at least one seizure arising from the typical and tapered condition, respectively. We could then proceed to detect states that only appear in the tapered condition, herein termed ‘taper-emergent states’. Indeed, we found 7 subjects with 30 seizures that contained taper-emergent states. On average per subject, there were 1.43±0.73 taper-emergent states, and 4.29±2.12 seizures that contained them. In all but one subject, these taper-emergent states recruited additional brain regions into seizure activity (Supplementary Section 5).

In supplemental analyses, we additionally investigated if these taper-emergent states preferentially involved specific brain regions (Supplementary Section 5), or if epilepsy type or ASM type were associated with taper-emergent states (see Supplementary Section 1). We found no strong evidence of either in our limited sample, but the descriptive summaries can be found in the supplementary. We also saw no evidence of epilepsy type (Supplementary Section 1), recording duration, tapering duration, or ASMs (Supplementary Section 1) being associated with the observation of taper-emergent states.

### Seizures containing taper-emergent states are substantially longer

**Figure 4:**
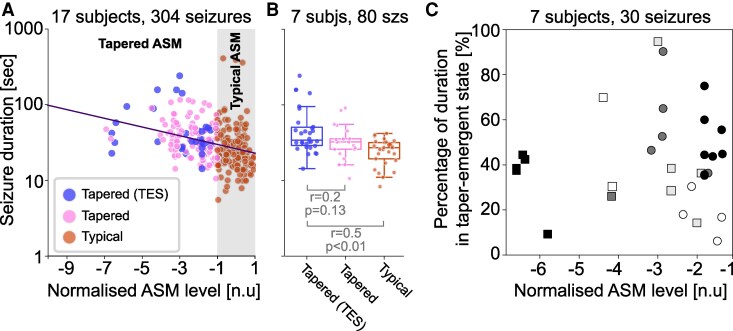
**Seizures containing taper-emergent states are longer, and the taper-emergent states themselves contribute substantially to the overall seizure duration.** (**A**) For the subset of subjects (n=17) with at least one seizure in the typical and tapered condition and their 304 seizures, we plotted seizure duration against normalized ASM levels. Note seizure durations shown here have systematic differences between subjects removed using a random offset LMM. Seizures occurring in the typical and tapered conditions are shown in orange and pink, respectively. Seizures containing taper-emergent states (TES) are highlighted in blue. Best fit of fixed effects from a LMM is shown as a solid line, with a slope β=−0.058, P<0.001. Each data point represents a seizure. (**B**) For the subset of subjects (n=7) that had at least one seizure containing taper-emergent states, we additionally show their seizure duration after removing subject-level difference for three categories as a box plot. The three categories are from left to right: Seizure during tapered condition, containing taper-emergent states (blue); Seizure during tapered condition, without taper-emergent states (pink); Seizure during typical ASM condition (orange). The rank sum test statistics and *p* values obtained from a Wilcoxon rank-sum test of *post hoc* analyses following mixed effects modelling in (**A**). Each data point represents a seizure. (**C**) For the subset of n=7 subjects and their 30 seizures that contained taper-emergent states, we scattered percentage of seizure duration in taper-emergent state against normalized ASM levels. Each data point is a seizure. The seven subjects are distinguished by colour and shape. subjs, subjects; szs, seizures.

Given these taper-emergent states, we investigated whether their presence impacted the duration of seizures. Using the 304 seizures across 17 subjects from the previous section, which ensured that each subject had at least one seizure arising from the typical and tapered condition, we first sought to confirm the correlation between seizure duration and normalized ASM levels as before (Fig. 4A). Using linear mixed-effects regression, we obtained β=−0.058, P<0.001 as the coefficient for ASM levels in predicting seizure duration. This translates to an approximate 12.5% increase in seizure duration for every unit reduction in normalized ASM levels, which is within our previous estimation bounds. Figure 4A additionally highlights those seizures containing taper-emergent states (as blue data points).

We then isolate the seven subjects that had seizures containing taper-emergent states, with all their 80 seizures in total (30 seizures with taper-emergent states and 50 seizures without). We compared log-durations between seizures with and without taper-emergent states (Fig. 4B), confirming that seizures containing taper-emergent states tended to be longer than those without, and substantially longer (Wilcoxon rank-sum r=0.485, P<0.001) than seizures occurring during the typical ASM condition. To better account for subject-level effects statistically, we also tested this relationship using a mixed-effect model (logDuration∼seizureCategory+(1|subject), where seizureCategory corresponds to the three categories in Fig. 4B) and found that both groups of seizures without taper-emergent states ($\beta_{\mathrm{Tap}}=-0.178,P=0.022$ for tapered condition, and $\beta_{\mathrm{Typ}}=-0.288,P<0.001$ for typical condition) are substantially shorter than seizures with taper-emergent states. Again, note that $\beta_{\mathrm{Tap}}=-0.178$ indicates a 66% decrease in seizure duration of seizures in the tapered condition without the taper-emergent states relative to seizures with. While our mixed-effects model revealed this differential reduction in seizure duration, a *post hoc* pairwise analysis using the Wilcoxon rank-sum test indicated no statistical difference. We ascribe these differing results to the fact that the mixed-effect model has the ability to account for subject-level variability while the Wilcoxon rank-sum test does not. In Supplementary Section 4, we also tested for the chance-level occurrence of these findings. We used 27 additional subjects who were not ASM tapered to establish null distributions and we found the increased duration of seizures containing taper-emergent states is above and beyond what would be expected by chance.

Finally, we determined the proportion of the seizure duration that was occupied by taper-emergent states in those 30 seizures and seven subjects that contained them. We found a median proportion of 38.5% across subjects and seizures, and these taper emergent states tended to occur later on in the seizure (Supplementary Section 5). However, the proportion of seizure duration taken up by the taper-emergent states was not correlated with ASM level across all subjects, but instead followed an all-or-nothing pattern in each subject (Fig. 4C).

### Tapering also prolongs specific dose-independent states

Finally, we tested if seizures without taper-emergent states were also prolonged with medication tapering. That is, we tested whether seizures consisting purely of dose-independent states were also prolonged with medication tapering. We included all seizures from the previous analysis (all subjects that had at least one seizure in each tapered and typical ASM condition) and removed any seizures containing taper-emergent states (resulting in 274 seizures across 17 subjects).

**Figure 5:**
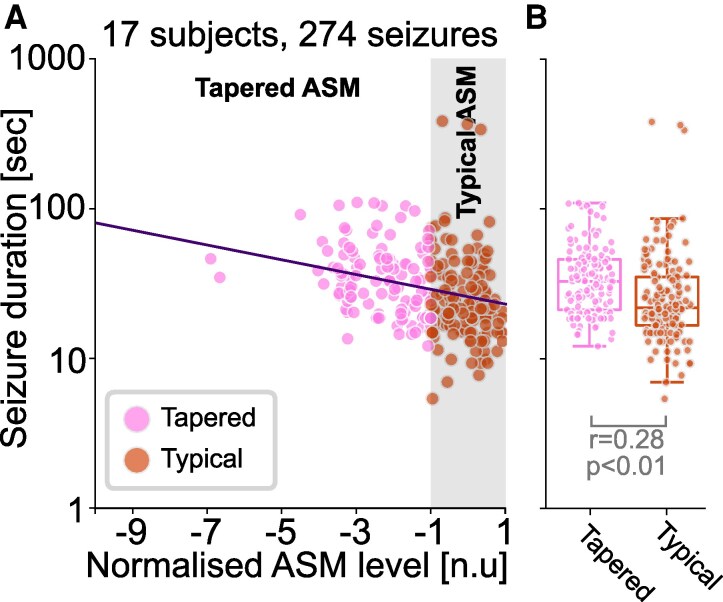
**Seizures without taper-emergent states are also prolonged with tapering.** (**A**) For the subset of subjects (n=17) with at least one seizure in the typical and tapered condition and their 274 seizures that did not contain taper-emergent states, we plotted seizure duration against normalized ASM levels. Note seizure durations shown here have systematic differences between subjects removed using a random offset LMM. Seizures occurring in the typical and tapered conditions are shown in orange and pink, respectively. Best fit of fixed effects from a LMM is shown as a solid line, with a slope β=−0.049, P<0.001. Each data point represents a seizure. (**B**) For the same subjects and seizures, we additionally show their seizure duration after removing subject-level difference as a box plot for tapered and typical ASM conditions. The rank sum test statistic and *P*-value obtained from a Wilcoxon rank-sum test of *post hoc* analyses following mixed effects modelling in A. Each data point represents a seizure.

Figure 5A demonstrates, as expected, that lower ASM-levels were associated with longer seizures (β=−0.049, P<0.001) in this subset. When comparing tapered versus typical ASM condition directly (Fig. 5B), we found that seizures occurring during ASM tapering were substantially longer than those occurring in the typical condition (Wilcoxon rank-sum: r=0.275, P<0.001). Both results indicate that ASM tapering also prolongs seizures without taper-emergent states.

It is important to note that this result does not mean that all seizures without taper-emergent states show the same seizure patterns and sequences, and are simply elongated, or ‘stretched out’ evenly with tapering. Rather, we found that tapering makes some states more common in seizures, and that only one or few states are elongated in each subject, resulting in an overall longer duration (Supplementary Section 6).

## Discussion

Our study provides new insights into how ASMs modulate seizure activity, revealing two distinct mechanisms. First, we found that ASM withdrawal can lead to the emergence of new seizure activity patterns in ∼40% of patients, which significantly prolonged the seizures they occur in. Second, even in the absence of these newly emergent states, lower ASM levels still resulted in prolonged seizure durations, with increases ranging from 12% to 224%, depending on the extent of tapering. Specifically, tapering does not appear to evenly stretch these seizures, but instead prolong specific states within them. These findings suggest that ASMs selectively suppress specific seizure patterns in an all-or-nothing manner, and selectively curtail other patterns in a dose-dependent manner. This dual role of ASMs in seizure control highlights their complex impact on brain activity and underscores the need for personalized dosing strategies to optimize epilepsy management.

Our findings have important implications for drug resistance in epilepsy. Previous research has suggested that ASMs shorten seizures, leading to the idea that they work by simply curtailing seizure activity into shorter durations. Our findings challenge this notion: We found that specific brain regions and seizure activity patterns emerge at certain ASM thresholds (Fig. 4), aligning with clinical observations that seizure suppression occurs abruptly—at a subject-specific level—rather than gradually with ASM titration.^24,25^ We propose that this all-or-nothing suppression reflects a *dynamic bifurcation*, where a gradual change in ASM levels triggers a sudden shift in seizure pattern. In contrast, the gradual shortening of certain seizure states represents a continuous, parameter-driven change. While the latter is expected in most (biological) systems, the former is rare—and linked to critical transition theory. This theory has been proposed for the onset of seizures,^26-28^ but our key contribution is demonstrating that these critical transitions also independently apply to certain seizure spread patterns with ASM tapering. Future studies should systematically investigate how different ASM types induce these mechanisms, considering variability across brain regions and epilepsy aetiologies. Ultimately, this could lead to a predictive model, where we observe the different seizure patterns arising in a given patient, and can predict the optimal ASM type and dosage needed to fully suppress all of them.

We demonstrate how ASM taper-emergent seizure dynamics may involve ‘new’ brain regions in seizure spread that were spared under typical ASM conditions. This highlights how a mapping of the interaction between these regions and ASM levels could provide important clues into seizure suppression, and drug-resistance. Further, a recent functional magnetic resonance imaging (fMRI) study of participants who had recently experienced their first seizure, Pedersen *et al*.^29^ revealed changes in widespread brain network function following ASM administration. These findings suggest that ASMs may impart their effects by engaging a spatial network of brain regions. In the context of our results, it highlights the question if specific brain networks experience one ASM mechanism more so than another. This question also needs to be answered in the context of the seizure semiology. In our study, the sample size was insufficient and the intracranial sampling was too subject-specific to test this. However, future work could test this in scalp EEG data, where we have more complete coverage of the entire brain. It would be both interesting to investigate if taper-emergent states preferentially invade new brain regions proximal to the seizure onset, but also if specific brain regions are differentially vulnerable to withdrawal of specific types of ASMs independent of onset location.

To provide some further interpretation of these seizure states, we highlight a particular area for future research. These seizure states correspond to specific activity patterns in specific brain regions. They will most likely correlate with seizure semiology states and networks,^30^ and could be formally tested in future work. Importantly, in some patients we may not be able to suppress all seizure states with ASMs, but may need to focus on some states that are more challenging, e.g. as they represent particular symptoms (loss of awareness or similar). Clinically, we know that even in drug-resistant patients, ASMs help suppress particularly severe seizures, most likely because they rescue critical function and—in the language of our work—effectively prevent certain seizure states from being accessed. Indeed, 60% of seizures with taper-emergent states had these states arise second or later in the observed states sequence (Supplementary Section 5). From this finding, we can infer that ASMs may involve altering seizure dynamical states to prevent propagation to broader regions rather than suppressing specific onset states. This behaviour is supported by existing evidence that ASMs prevent seizure spread,^31^ as well as clinical practice wherein ASM tapering enables pre-surgical evaluation to determine seizure onset zone for example.^32^ Future work should also test if particular seizure states influence the post-ictal state and recovery. If so, these states could become targets for ASM treatment. In parallel, we should also investigate if ASMs have a direct effect on the post-ictal recovery independent of seizure states.

Furthermore, we speculate if a similar dual mechanism is at play for interictal brain activity states. Patients on ASMs often experience cognitive impairments as a result of the medication,^33^ and it would be interesting to assess if this may be due to certain brain states suddenly becoming inaccessible. Similarly, ASMs are thought to also impact sleep and sleep architecture.^34^ We speculate that the dual mechanism shown here has relevance for understanding the relationship of certain ASMs and sleep, independent of seizure occurrence. Finally, ASMs are also used for other conditions such as mood disorders and migraines. Future work should investigate the relevance of either mechanism in those conditions.

### Limitations and future work

Our study was performed on a retrospective, observational, small sample. This means that questions around causality and covariates could not be answered definitively. For example, we found no evidence of any specific types of epilepsy or ASM influencing our results. This does not mean that there is no effect, but simply that we require larger samples to investigate the size and direction of these covariates and interactions.

We used electrographic seizure duration here, as our whole analysis rested on the electrographic data. However, clinical seizure duration may differ and future work should explore implications thereof.

Our patient cohort were all invasively implanted with iEEG, meaning the start of their recording will include some effects of anaesthesia in addition to any ASM effects. We acknowledge that this post-implant effect may confound our observations between seizure states and ASMs, although our supplemental analysis using non-tapered subjects alleviates this concern somewhat.

Our work focused on broad and recurring seizure EEG patterns as states, meaning that we did not investigate seizure onset specifically. Future work has to investigate in what way seizure onset changes with ASM tapering, although currently literature^35^ and clinical practice suggest and assume minimal changes.

We were also not able to directly investigate the effect of sleep, as this data was not consistently recorded in our retrospective data. Our circadian analysis suggests that there is a subtle effect across the cohort in terms of seizure duration, which we account for in our main results (see Supplementary Section 3). However, any future work with larger cohorts should account for sleep and other chronobiological effects (such as multidien cycles), as we know seizures and seizure states are influenced by these both in occurrence and duration.^11,36-38^ We suggest future studies using longer recordings within the same subject, and with a much longer observation period on stable ASMs would be best to create adequate baselines to account for both transient effects (e.g. anaesthesia) and cyclic effects (e.g. multidien cycles).

Finally, our study investigated a cohort of drug-resistant patients on poly-therapy. The mechanisms we highlight here may not generalize to the more frequently-encountered cohort: patients who are newly-diagnosed and are slowly introduced to their first medication. Initial drug resistance presents in over 30% of such subjects and perhaps a longitudinal study (using scalp-EEG or subcutaneous EEG) in this setting with our analytical approaches would be best to present a more holistic picture of ASM mechanism.

## Supplementary Material

fcag088_Supplementary_Data

## Data Availability

Anonymized seizure state data and ASM intake schedule, along with analysis code, is available on GitHub: https://github.com/cnnp-lab/2025_ASM-SzState_GMB.
