# Supplementary material for: Dual mechanism of anti-seizure medications in controlling seizure activity
Source: Brain Commun. 2026 Mar 16;8(2):fcag088. doi: 10.1093/braincomms/fcag088 (PMC13023363; doi:10.1093/braincomms/fcag088)
Supplement: fcag088_Supplementary_Data [file fcag088_supplementary_data.pdf]

Supplementary Sections

1 Subjects details

Table 1 provides details regarding demographics, iEEG recordings, seizure types and tapered ASMs. We provide these details for the complete dataset, the reduced dataset, and the seven individuals with taper-emergent states. Remember in the reduced dataset, we selected subjects who had at least three seizures overall, with at least one in each of the tapered and typical ASM conditions. In the seven individuals with taper-emergent states, we did not find any single medication specific to these subjects.

	Complete	Reduced dataset	"Taper-emergent"
No. subjects	28	17	7
Age (mean±SD)	33.1±8.2	33.0±7.3	33.9±8.2
Sex (F%)	46.4%	29.4%	0.0%
Epilepsy (TLE%)	57.1%	64.7%	57.1%
Side (Left%)	39.3%	35.3%	42.9%
No. iEEG contacts (mean±SD)	86.9±23.2	82.1±20.2	91.4±22.7
No. ROIs (mean±SD)	19.6±5.5	18.5±5.0	18.0±2.9
Days of recording (mean±SD)	12.2±2.7	12.3±3.2	13.1±2.3
No. seizures	457	308	80
Sub-clinical	45.7%	43.5%	36.2%
Focal	52.1%	54.3%	60.0%
SG	0.7%	0.6%	2.5%
Other	1.5%	1.6%	1.2%
No. regular ASM (mean±SD)	3.2±1.0	3.2±1.0	3.1±0.3
No. tapered ASM (mean±SD)	2.3±0.7	2.3±0.7	2.7±0.5

Supplementary Table 1: Summary of subject data used in the analysis. SG: secondarily generalized Seizures. SD: Standard Deviation, F: Female, TLE: Temporal Lobe Epilepsy, ROI: Region Of Interest, ASM: Anti-Seizure Medication, iEEG: Intra-Cranial Electroencephalography

Subject ID	Recording duration	Typical condition	Tapered condition
S42	8.58	4.67	3.92
S44	8.92	5.88	3.04
S46	8.5	6.71	1.79
S48	8.38	3.83	4.54
S50	4.96	2.13	2.83
S52	8.92	6.08	2.83
S54	10	4.67	5.33
S56	12.13	5.21	6.92
S58	9	0.33	8.67
S60	8	2.42	5.58
S62	11.08	4.92	6.17
S64	5.25	0.08	5.17
**S66**	**10.79**	**2.83**	**7.96**
**S68**	**11.25**	**5.83**	**5.42**
S70	8.54	3.46	5.08
S72	9.42	4.46	4.96
**S74**	**5.04**	**0.75**	**4.29**
**S76**	**5.38**	**0.79**	**4.58**
S78	5.54	0.63	4.92
S80	4.17	0.04	4.13
S82	18.71	4.67	14.04
**S84**	**5.92**	**3.13**	**2.79**
**S86**	**6.17**	**5.17**	**1**
S88	8.17	3.46	4.71
S90	12.58	5.92	6.67
S92	12.42	8.42	4
S94	9.88	6.25	3.63
**S96**	**6.58**	**3.21**	**3.38**

Supplementary Table 2: Summary of subject durations for recordings, and tapered and typical periods used in the analysis in days.

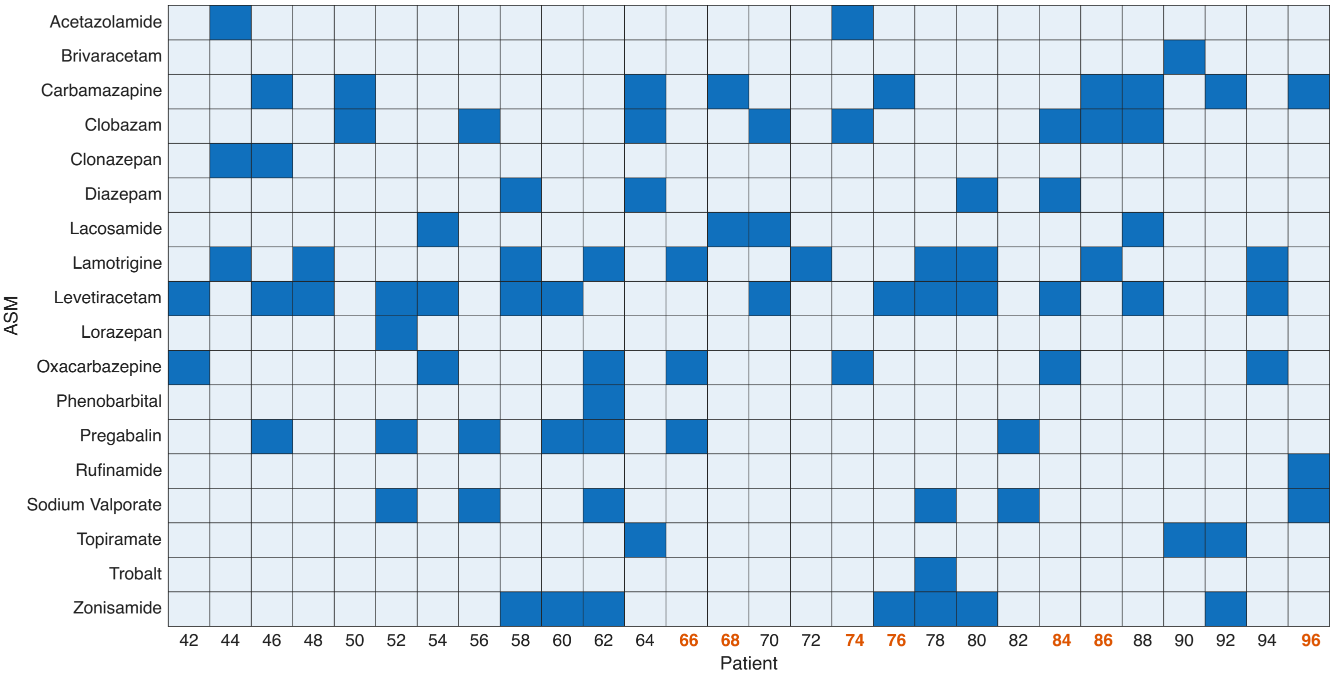

Supplementary Figure 1.1: Summary of ASMs that patients were taking. Except for patient 72, all patients were on at least 1 ASM. Blue denotes the ASM administered to the patient; and white denotes not taking the ASM. Patient IDs indicated in orange color and bold font represent patients in whom we observed taper-emergent states.

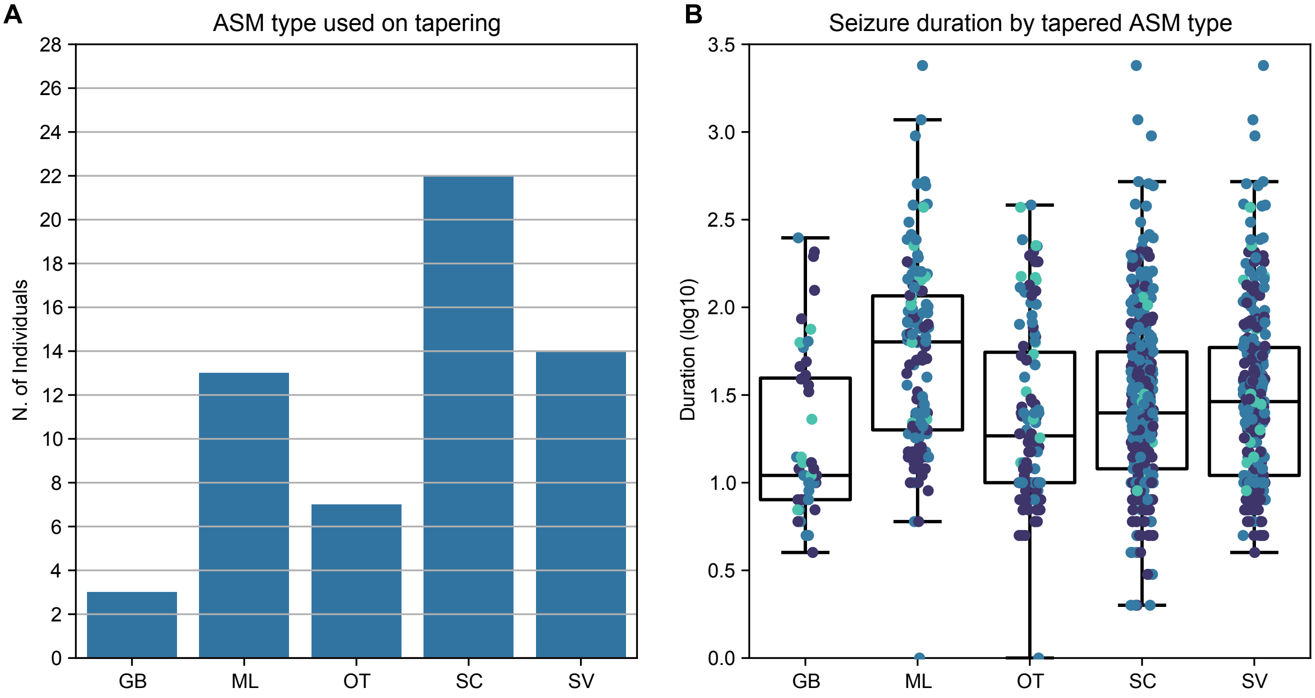

Supplementary Figure 1.2: Summary of ASM types administered during tapering, including seizure duration responses for each ASM type. A: ASM type during tapering. **B**: Seizure duration grouped by tapered ASM type. Each data-point is colour-coded by seizure type (dark blue = typical seizures, blue = tapered seizures, light blue = tapered seizures with taper-emergent state). Note one seizure can appear multiple times in this plot if the patient had multiple ASM types tapered. Type of ASM related to the target; GB: GABA receptor, ML: multitarget, OT: other medication (low representation), SC: sodium channels, SV: SV2A receptor.

2 Algorithmics details on seizure states identification

2.1 Pre-ictal period noise removal

As stated on *Methods, Identification of seizure states*, we used the pre-ictal period (commencing 120 seconds prior to seizure onset) as a baseline to z-score band-power during the ictal period. We excluded potential preictal noise by removing any preictal time-window with a Median Absolute Deviation (MAD) score exceeding a threshold of five. For each combination of region and frequency band, we z-scored ictal values against the preictal distribution. Any negative ictal z-scores were excluded (i.e., replaced with 0) as we were only interested in increases in activity compared to the preictal segment. Note that negative ictal z-scores most likely represent elctrodecrement events, which should be treated differently to seizure activity, and we reserve their investigation for a separate paper.

2.2 Reducing observation for Non-negative Matrix Factorisation (NMF)

We aimed to avoid the NMF algorithm over-prioritising states that a seizure stays in for a extended period of time. This was achieved by clustering time points in the data matrix X if they had a correlation coefficient of more than 0.8. We then kept only one time point in each cluster to produce a reduced matrix rX with dimensions $(n_{regions} * n_{bands}) \times n_{T_{reduced}}$, where $n_{T_{reduced}}$ is the number of clusters below 0.8 in correlation coefficient.

We applied NMF as described in the main text to this reduced data matrix to obtain: $rX \approx W \times rH$ and reconstructed the 'actual' H matrix by $H = WnX$, where X is the original data matrix. We essentially used the W matrix from the reduced data matrix to reconstruct what H must look like given the original data matrix X. Note this H matrix is not guaranteed to be non-negative now, but will contain small negative numbers, which we will understand as "noise" and leverage later.

2.3 NMF optimisation

When running the NMF algorithm, we selected a value for the number of components (n_c) by testing a range of values from 2 to $\sqrt{n_{T_{reduced}}}$ and finding the value for n_c that produced the most stable components. This was tested following the steps shown in [1, 2]. For each value of n_c the NMF algorithm was ran 30 times and an average connectivity matrix C was produced. We then calculated the dispersion coefficient ρ using:

$$\rho = \frac{1}{n^2} \sum_{i=1}^{n} \sum_{j=1}^{n} * \left((C_{ij} - \frac{1}{2} \right)^2 \tag{2.1}$$

We then selected the value for n_c that maximised ρ.

2.4 Definition of null state

As explained in methods section 2.4, each time window is assigned to the most strongly expressed component to create the states analysed in this paper. However if no component is expressed strongly, a time point will be assigned to a "null" state. To define the threshold for low expression, we found an estimation of the "noise" in H. We leveraged the fact that the H matrix contains small negative numbers, which we assume to be "noise" from the NMF reduction. We mirror these negative numbers to give us a symmetric distribution around zero, derive the standard deviation of said distribution, and set the threshold of the null state at 3 times the standard deviation. We are effectively saying that – assuming the small negative and positive numbers in H are noise – we only deem a time window to be a seizure state if it exceeds this noise threshold. Note that we are most likely ignoring small seizure activity limited to few channels and frequency bands (e.g. at the start of seizures). Such onset patterns would most likely fall below the threshold and be classified as a null state. Future work wishing to investigate onset patterns specifically will have to modify our algorithm. In this work, we were not interested in onset patterns, but were only investigating seizure propagation patterns.

3 Hierarchical Models to determine relationship between seizure duration and ASM levels

To determine the relationship between normalised ASM levels and seizure duration, a linear mixed-effects model (LMM) was used:

$$\begin{aligned} \textit{log-duration} \sim & \beta_0 \\ & + \beta_1 \textit{ASM-level} \\ & + \beta_2 sin(TOD) + \beta_3 cos(TOD) \\ & + \beta_4 sin(12hTOD) + \beta_5 cos(12hTOD) \\ & + (1|subject) \end{aligned} \tag{3.1}$$

Duration on log10 scale (*log-duration*) was predicted using a fixed-effect on normalized ASM plasma concentration levels (ASM_lvl) and a random intercept for each subject (*subject*). To account for circadian and 12 hour fluctuations fixed-effect on time of day (TOD) was included as a circular variable. Sine ($sin(TOD)$) and cosine ($cos(TOD)$) of time of day represent circadian rhythms, while $sin(12hTOD)$ and $cos(12hTOD)$ represent the ultradian rhythm 12 hour cycle. One reason for including the 12 hour cycle is to factor out any effects of ASM intake which are 12-hourly (morning and evening intake).

This model was applied in three scenarios: 1) the complete dataset of 28 individuals and 457 seizures, 2) reduced dataset for seizure states analysis of 17 individuals and 308 seizures, 3) same 17 individual, but without seizures containing taper-emergent states resulting in 274 seizures. Coefficients and p-values for these three cases are reported on Table 3 including fixed-effects of ASM plasma levels.

3.1 Interaction of ASM levels and time of day

We next sought to test if excluding the time of day (both 24h and 12h) effects impacts our findings of ASM levels. To this end, we fitted a new model only featuring the ASM levels and the random-

Dataset [subj., sz.]		ASM-lvl	sin(TOD)	cos(TOD)	sin(12hTOD)	cos(12hTOD)
Full Dataset [28, 457]	β	-0.032	-0.057	-0.035	-0.026	-0.015
	p	<0.001	0.008	0.133	0.252	0.474
States Study [17, 304]	β	-0.058	-0.049	-0.012	-0.011	-0.041
	p	<0.001	0.044	0.644	0.664	0.086
Typical Seizures [17, 274]	β	-0.049	-0.058	-0.029	-0.028	-0.049
	p	<0.001	0.016	0.296	0.285	0.044

Supplementary Table 3: Summary of the hierarchical models in eqn. 3.1 fitted to generate the main results of this study showing the fixed-effect coefficients (β) and p-values (p), including ASM levels and the circular variables representing the circadian (time of day - TOD) and 12h (12hTOD) effects.

effect of subject: [$log\text{-}duration \sim \beta_0 + \beta_1 ASM\text{-}level + (1|subject)$]. Figure 3.1 mimics Figure 4A with the modified model, showing no substantial change on the new model. β_1 shows a difference between models around 0.001 (representing a difference of 0.2% on increase rate) with similar p-values (< 0.001).

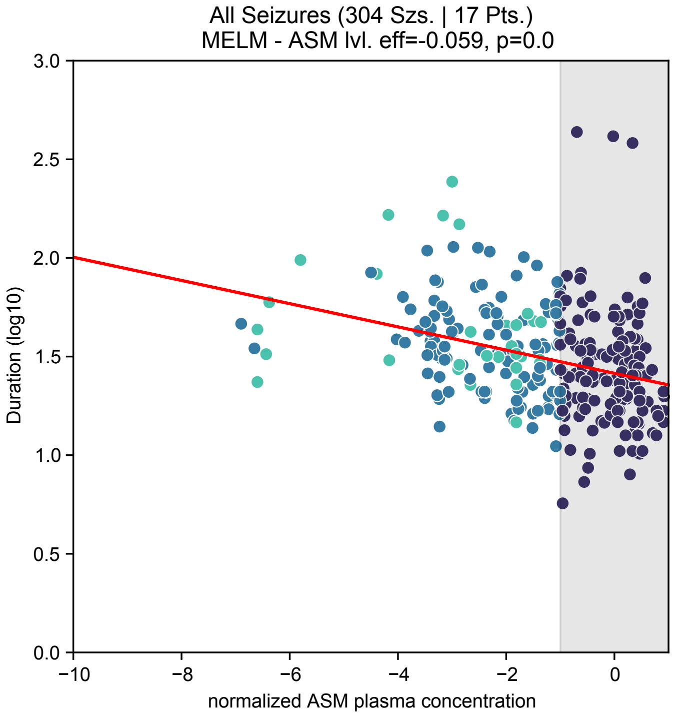

Supplementary Figure 3.1: Exclusion of time of day does not distort the effect of ASM in predicting seizure duration. We reproduced Figure 4A without the time of day circular variables. For the subset of subjects (n=17) with at least one seizure in the typical and tapered condition and their 304 seizures, we plotted seizure duration after removing systematic differences between subjects [using a random offset linear mixed-effects model (LMM)] against normalised ASM levels. Compared to the LMM presented in the main paper (Eqn. 3.1), this new model does not include fixed-effects related to 24h and 12h rhythms. Seizures occurring in the typical and tapered conditions are shown in navy and blue, respectively. Seizure containing taper-emergent states are highlighted in cyan. Best fit of fixed effects from a LMM is shown as a solid red line, with a slope $\beta = -0.059$, $p < 0.001$. Each data point represents a seizure. Szs: seizures; Pts: points; lvl: level.

4 Testing for chance-level properties of taper-emergent states

In this section, we will examine if the properties of taper-emergent states and their associated seizures in our data could have arisen by chance. To answer this question, our rationale was to generate a null distribution in a range of properties for each of the seven subjects that showed taper-emergent states.

For each of the seven subjects, we applied their ASM tapering profile to all $n = 27$ non-tapered subjects and their seizures. In other words, we can generate 27 "null" simulations of ASM tapering for each subject and are able to obtain "pseudo-typical" and "pseudo-tapered" seizures, and therefore also "pseudo-taper-emergent states". For each of the seven subjects, we then compared their actual seizure and seizure state properties against these pseudo seizure and state properties. The 27 non-tapered subjects therefore served as a control cohort for each of the seven subjects.

First, we focused on our main finding that seizures with taper-emergent states are longer in duration than other seizures, and particularly other typical-ASM dose seizure in the same subject. For each of the seven subjects, we computed the difference in seizure duration of seizures with taper-emergent states and those without; we then applied the same calculation to the pseudo-seizures, which generated a null distribution of the same property and used this to determine where the observed value from the patient falls in this null distribution (Figure 4.1). We can observe that in five of the seven patients, the actual observations were more extreme relative to the pseudo distributions. We can interpret this as the seizure duration difference between seizures containing taper-emergent states vs. all other seizures being different to chance-level expectations. Surprisingly, the null distributions sometimes show a substantially larger difference than the actual observation. This most likely reflects other modulations in the 27 non-tapered subjects (also see later discussion).

Second, we refined the previous test to compare seizures with taper-emergent states *vs.* typical-ASM dose seizures, which showed the strongest effects in our main results. We again applied the

same analysis to the pseudo seizures as above, and observe that in all seven patients, the actual observations were larger relative to the pseudo distributions (Figure 4.2). We can interpret this as the duration of seizures containing taper-emergent states being longer than typical-ASM dose seizures even compared to chance-level expectations.

Third, we also investigated if the relative duration of the taper-emergent states we observed in each patient could have arisen by chance (Figure 4.3). We again observed that in five of the seven patients (not the same five), the actual observations were more extreme relative to the pseudo distributions. We can interpret this as the relative contribution of the taper-emergent state to overall seizure duration being different to chance-level expectations. Surprisingly, again, the null distributions sometimes show a substantially larger contribution than the actual observation. This most likely reflects other modulations in the 27 non-tapered subjects (also see later discussion).

In general, we observe that the properties of taper-emergent states in patients relative to null distributions were more extreme, meaning that the properties of the taper-emergent states in the seven subjects were most likely not "by chance". However, we caution against drawing strong conclusions from these results. There are several issues to consider with using non-tapered patients to generate chance-level null distributions. The non-tapered patients are most likely not directly comparable with the tapered patients in their drug-resistance mechanism, and seizure state modulation. These modulations can be seen in Figure 4.1 and Figure 4.2, in the fact that the null distributions are not centred around zero.

These non-tapered patients also had more frequent seizures even on full medication, and the seizures they had were also more severe; whereas the tapered patients tended to have more subclinical seizures before and after tapering. Non-tapered subjects were generally also recorded for a shorter amount of time, meaning that not all 27 non-tapered subjects could be used for every one of the seven patients with taper-emergent states. Thus, comparison across the seven subjects is also difficult.

We suggest future work investigate a better "control" scenario. Ideally, each patient should be their

own “control”, which also allows us to account for subject-specific modulations such as circadian and multidien cycles in their seizures[3] and seizure states[4]: Future work could use long-term recordings that include a short ASM taper period. In this manner, we can observe more typical seizures, and the null distribution could be sampled over a period of static ASM levels.

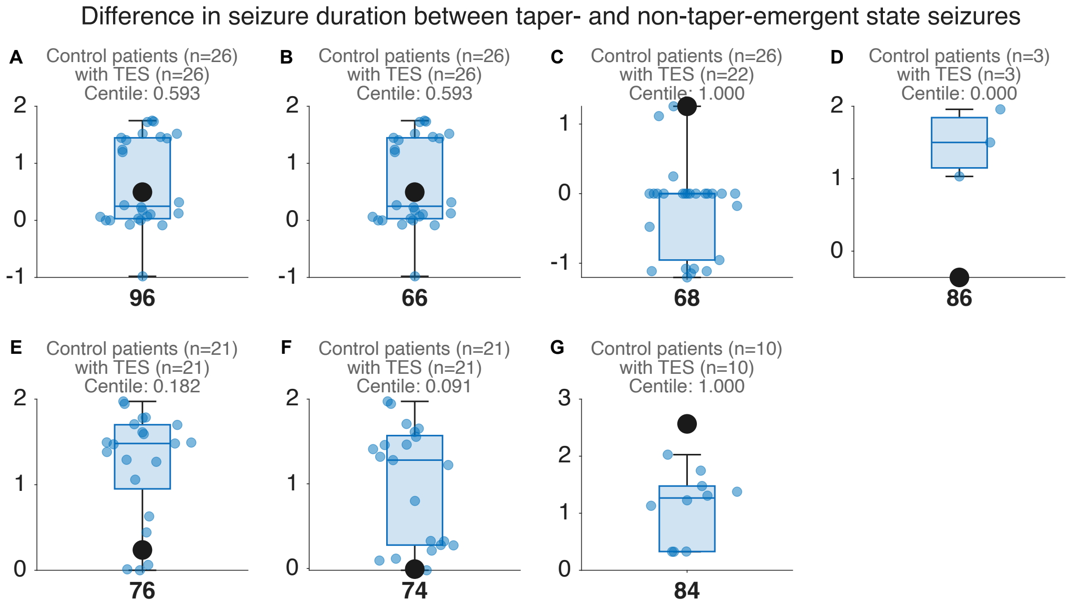

Supplementary Figure 4.1: Difference in log10 seizure duration between seizures containing taper-emergent states and all other seizures. A-G: Each panel represents one of the seven subjects that showed taper-emergent states (TES). Y-axis shows the average difference in log10 seizure duration, i.e. the average ratio between seizures containing taper-emergent states and all other seizures. Each black data point represents the actual observation in that patient. If multiple TES were observed in a given patient, the black data point shows the average. The blue boxplot and data points show the null distribution derived from non-tapered patients and their seizures. Note, not all 27 non-tapered patients could be used for every one of the seven patients with taper-emergent states, the actual number of controls used is stated at the top of each panel. Also, a TES was not always found in all controls, and the number of controls with TES is stated at the top of each panel, which is also the number of blue data points. Other than patients 96 and 66, all others show a more extreme observation relative to their null distributions.

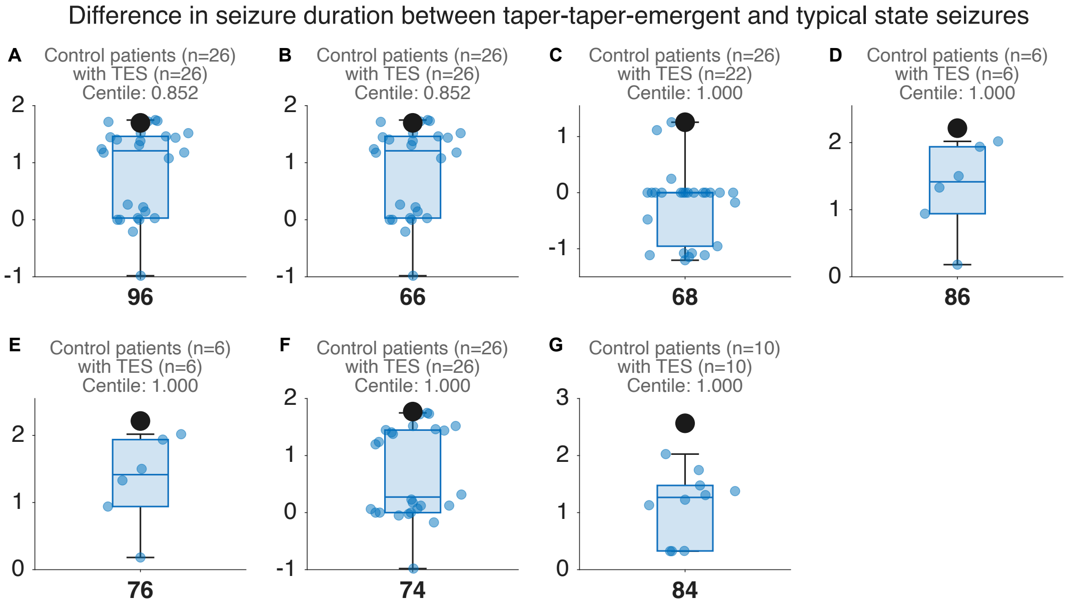

Supplementary Figure 4.2: Difference in log10 seizure duration between seizures containing taper-emergent states and typical-ASM dose seizures. A-G: Each panel represents one of the seven subjects that showed taper-emergent states (TES). Y-axis shows the average difference in log10 seizure duration, i.e. the average ratio between seizures containing taper-emergent states and typical-ASM dose seizures. Each black data point represents the actual observation in that patient. If multiple TES were observed in a given patient, the black data point shows the average. The blue boxplot and data points show the null distribution derived from non-tapered patients and their seizures. Note, not all 27 non-tapered patients could be used for every one of the seven patients with taper-emergent states, the actual number of controls used is stated at the top of each panel. Also, a TES was not always found in all controls, and the number of controls with TES is stated at the top of each panel, which is also the number of blue data points. All patients show a more extreme observation relative to their null distributions.

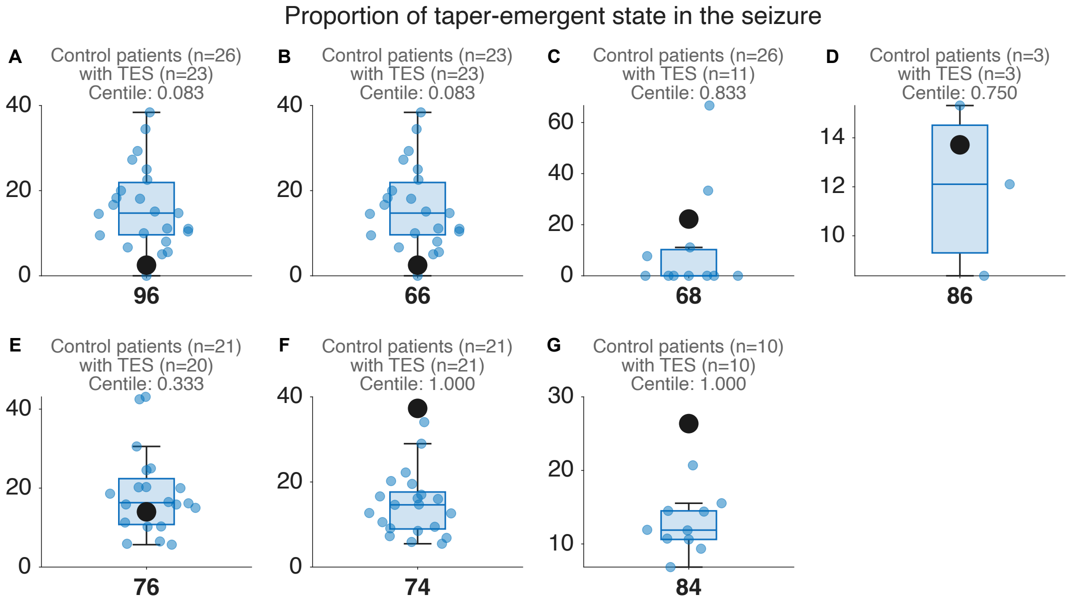

Supplementary Figure 4.3: Proportion of taper-emergent state in the seizure. A-G: Each panel represents one of the seven subjects that showed taper-emergent states (TES). Y-axis shows the proportion the TES takes up in their seizure duration as percentage. Each black data point represents the actual observation in that patient. If multiple seizures with TES were observed in a given patient, the black data point shows the average. The blue boxplot and data points show the null distribution derived from non-tapered patients and their seizures. Note, not all 27 non-tapered patients could be used for every one of the seven patients with taper-emergent states, the actual number of controls used is stated at the top of each panel. Also, a TES was not always found in all controls, and the number of controls with TES is stated at the top of each panel, which is also the number of blue data points. Other than patients 76 and 86, all others show a more extreme observation relative to their null distributions.

5 Further characterizing taper-emergent states

We performed additional analyses characterizing taper-emergent states. As stated in our methods, seizures with taper-emergent states were longer compared to seizures without them ($p < 0.001$).

We also studied the sequence of states within taper-emergent seizures (Figure 5.1 left) comparing the first appearance of each state type. In 60% of seizures with taper-emergent states, these appeared as the second or later in the sequence of states.

Figure 5.1 right further shows that taper-emergent states substantially contribute to overall seizure duration, with a median across these seizures (N=30) of 38.5%. Moreover, this contribution was not correlated with ASM level across all subjects (Figure 5.2), but followed more a all-or-nothing pattern: they emerge suddenly at a subject-specific normalised ASM level and can contribute to between 20-80% of the overall seizure duration.

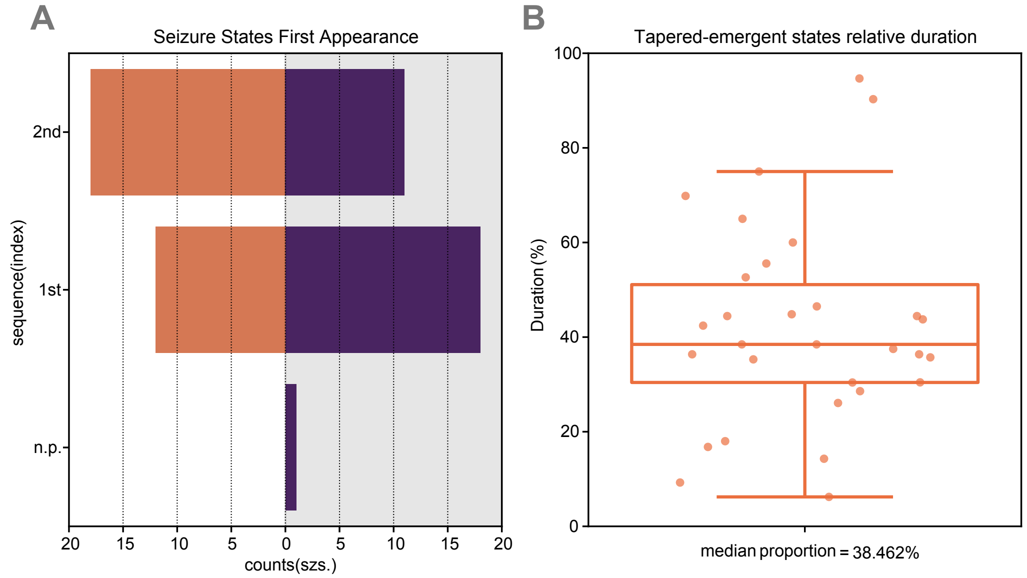

Supplementary Figure 5.1: Taper-emergent states have a substantial contribution to seizure duration predominately during later stages of the seizure. **A**: Study of states sequence comparing taper-emergent states (orange) and dose-independent states (purple) on seizures containing taper-emergent states (30 seizures, 7 individuals). **B**: the number of seizures where the state type is present the first (1st), the second (2nd) or it is not present (n.p.). Each data point represents a seizure.

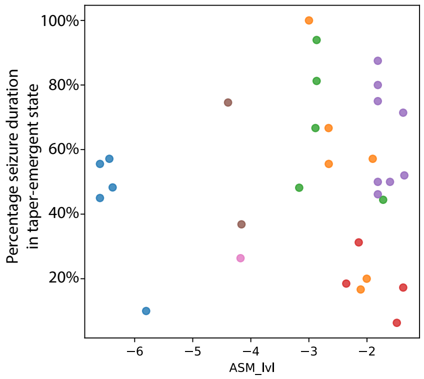

Supplementary Figure 5.2: Contribution of taper-emergent states to overall seizure duration plotted against ASM levels. Each data-point corresponds to a seizure (30 seizures) colour-coded by individuals (n=7).

Next, we compared signal amplitude and spatial spread between taper-emergent and dose-independent states. As a result of the NMF, the data matrix for each individual ($X = (nROI * nFrq) \times nEpoch$) was decomposed into matrices W ($(nROI * nFrq) \times nStt$) and H ($nStt \times nEpoch$). The W matrix can be interpreted as levels of band power involvement (abnormal relative to baseline, i.e. involved in seizure) of each state across ROIs and frequency bands. To identify which ROIs were recruited by each state, the following steps were followed:

1. W across frequency bands for each ROI is squared and summed across frequency bands for each ROI.
2. The resulting number is divided by the number of ROIs.
3. Recruited ROIs are identified if the final number exceeds 1.

As each state in W is of euclidean norm 1, the above steps measure above average involvement of each channel.

From this, we identified ROIs that were recruited by each state type (taper-emergent and dose-independent), as well as which ROIs were exclusively recruited by taper-emergent states. Taper-emergent and dose-independent states do not show any mayor differences on the number of recruited ROIs (Figure 5.3A). However, we found at least one additional ROI is exclusively recruited

during taper-emergent states in most subjects (6/7). With the recruited ROIs identified, the amplitude during taper-emergent state can be compared.

Amplitude for each ROI was obtained as the sum of the squares of the band power for each frequency bands. Note that amplitude is to be interpreted as the abnormality z-score of the ictal log band power compared to pre-ictal. Then, for each state we averaged across recruited ROIs and active time-window (Figure 5.3B). Additionally, the average amplitude for ROIs exclusively recruited by taper-emergent states is estimated. Taper-emergent states show a higher amplitude compared to dose-independent states in some subjects (4/7), despite ROIs exclusively recruited by taper-emergent state showing lower amplitude than the average.

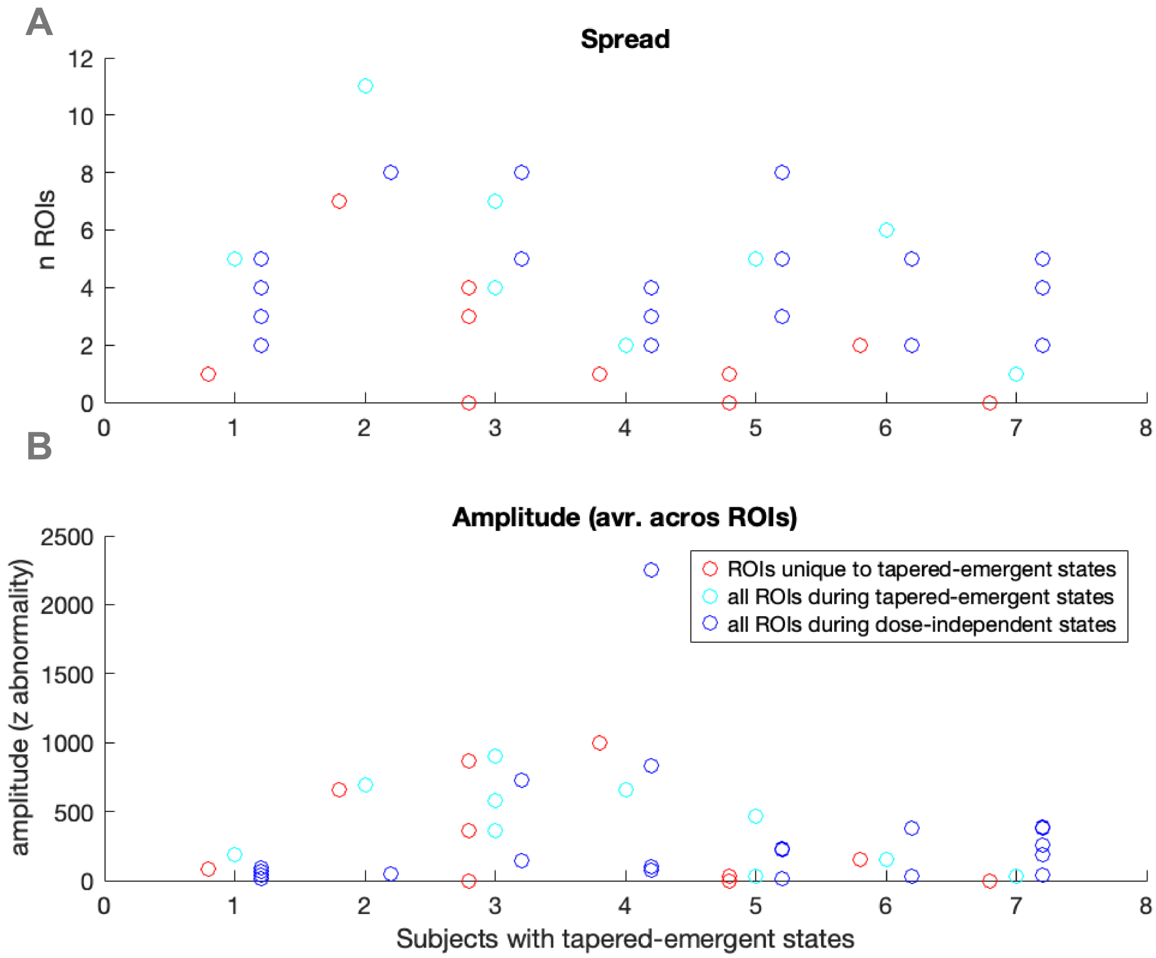

Supplementary Figure 5.3: Taper-emergent states recruit additional ROIs, and show higher amplitude than dose-independent states. For both panels each data-point represents a state. X-axis numbers are separate subjects ($n = 7$). Dose-independent states are shown in navy blue. Taper-emergent states are presented in cyan. Red data points show regions that were exclusive to taper-emergent states. **A**: Seizure state spread measured as the number of ROIs with involvement in said state. **B**: Average amplitude (z-score relative to preictal) of the ROIs for each state.

To summarise the complex data of the number of ROIs involved in a different way, we also plotted the maximum number of ROIs invaded at any given time in a seizure, averaged across seizures in Figure 5.4. We split the seizures up into three different categories, namely seizures that occurred during tapering and contained taper-emergent state(s) (TES); seizures that occurred during tapering and did not contain any taper-emergent state; and seizures that occurred on the typical ASM dosage. We observed that on average across subjects, the seizures with TES were the most wide-spread seizures. However, individual subjects could also have some very wide-spread seizures

during typical ASM dosage. Future work is warranted, where the mechanism of ASM tapering is specifically investigated in terms of spatial patterns during recruitment. However, we suggest this is best performed with scalp EEG data where the spatial coverage is more complete. In our data, the number of ROIs implanted is an obvious covariate making direct comparison across subjects difficult.

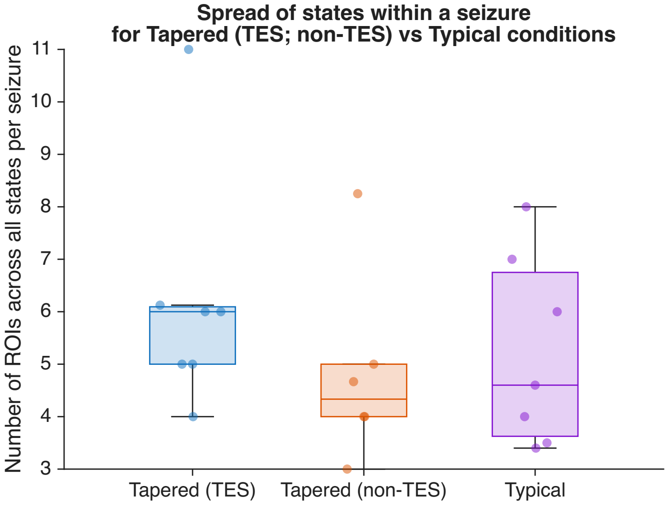

Supplementary Figure 5.4: Maximum number of ROIs invaded at any given time in a seizure, averaged across seizures in each subject in three different conditions. Comparing the maximum spread (across all states) of seizures by number of ROIs. Each data point represents a subject's average across its seizures in a given category.

Finally, we also present the raw data of each of the seven subjects that had TES. In Figure 5.5, we show for each subject (column) the ROIs that were implanted, and also how many times each ROI (rows) was observed in a taper-emergent state.

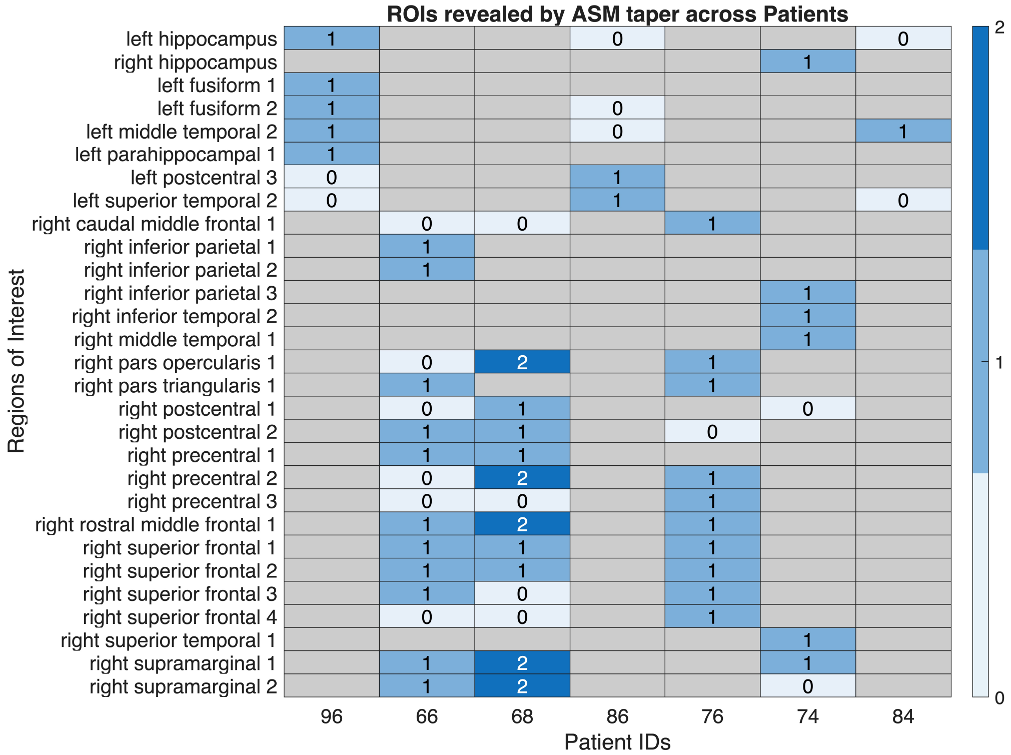

Supplementary Figure 5.5: Summary of ROIs involved in taper-emergent states. A heatmap color-coded based on the number of ROIs involved in taper-emergent states. The grey-coded cells represent non-implanted regions for the respective subjects $n = 7$. We have omitted ROIs where none of the subjects had any implantation.

6 Further characterizing dose-independent states

We further characterize seizures only composed of dose-independent states (274 seizures, 17 individuals). As a reminder, dose-independent states appear in both conditions of typical and tapered ASM. In other words, we have removed any seizures that contain taper-emergent states for this analysis.

Analysing the number of states in these seizures (Figure 6.1A), there is no difference between seizures occurring during typical and tapered conditions.

Given that multiple dose-independent states were present in each seizure (average: 2.5, standard deviation: 1.2), the contribution of these states to the total seizure duration was studied. We assessed if all dose-independent states in a seizure were contributing equally to total seizure duration. For every state we applied Spearman Correlation between its duration and the total seizure duration (Fig. 6.1B) across all seizures in each subject. We identified at least one state with a correlation coefficient above 0.7 and $p < 0.05$ in most subjects, but by no means were all state durations highly correlated with overall seizure duration. This indicates that the prolonged seizure duration with ASM tapering is by no means "stretching" all states equally (we would otherwise see perfect 1:1 correlations in all states). Rather, in most subjects, one or few states are stretched in line with overall seizure duration. In other subjects, there is no consistent pattern.

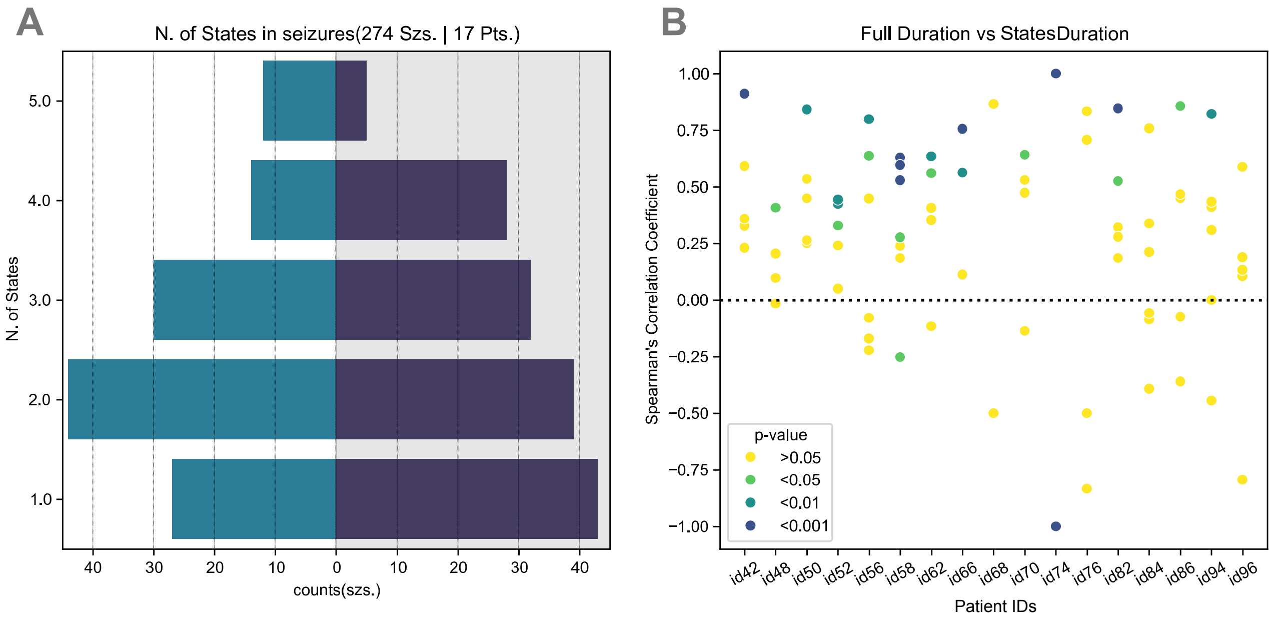

Supplementary Figure 6.1: The increase in duration of seizures without taper-emergent states is not homogeneously distributed amongst the states. This study is applied to all seizures without taper-emergent states, i.e. solely consisting of dose-independent states (274 seizures, 17 individuals). **A**: Distribution of the number of states in each seizure, summarised across all seizure for the typical (right, navy colour) and tapered (left, blue colour) condition. **B**: Correlation between individual state duration and the overall seizure duration. Each point represent a state, sorted by subjects in columns. The y-axis indicates the Spearman's Correlation Coefficient between state duration and seizure duration, and the colour of each data point is based on p-values.

Supplementary References

[1] Jean-Philippe Brunet et al. "Metagenes and molecular pattern discovery using matrix factorization". In: *Proceedings of the National Academy of Sciences* 101.12 (Mar. 2004), pp. 4164–4169. DOI: 10.1073/pnas.0308531101. (Visited on 03/13/2025).

[2] Hyunsoo Kim and Haesun Park. "Sparse non-negative matrix factorizations via alternating non-negativity-constrained least squares for microarray data analysis". In: *Bioinformatics* 23.12 (June 2007), pp. 1495–1502. ISSN: 1367-4803. DOI: 10.1093/bioinformatics/btm134. (Visited on 03/13/2025).

[3] Philippa J Karoly et al. "Cycles in epilepsy". In: *Nature Reviews Neurology* 17.5 (2021), pp. 267–284.

[4] Gabrielle M. Schroeder et al. “Chronic intracranial EEG recordings and interictal spike rate reveal multiscale temporal modulations in seizure states”. In: *Brain Communications* 5 (5 Aug. 2023).
